# Chronic Lung Disease Following Neonatal Extracorporeal Membrane Oxygenation: A Single-Center Experience

**DOI:** 10.3389/fped.2022.909862

**Published:** 2022-07-08

**Authors:** Alba Perez Ortiz, Anna Glauner, Felix Dittgen, Thalia Doniga, Svetlana Hetjens, Thomas Schaible, Neysan Rafat

**Affiliations:** ^1^Department of Neonatology, University Children’s Hospital Mannheim, University of Heidelberg, Mannheim, Germany; ^2^Department for Medical Statistics and Biomathematics, Medical Faculty Mannheim, University of Heidelberg, Mannheim, Germany

**Keywords:** chronic lung disease, extracorporeal membrane oxygenation, respiratory failure, neonatal lung disease, congenital diaphragmatic hernia

## Abstract

**Objective:**

To assess the incidence and severity of chronic lung disease (CLD) after neonatal extracorporeal membrane oxygenation (ECMO) and to identify factors associated with its development.

**Methods:**

A retrospective observational study in a neonatal ECMO center was conducted. All neonates who received support with ECMO in our institution between January 2019 and October 2021 were included and their pulmonary outcome was investigated.

**Results:**

A total of 91 patients [60 with congenital diaphragmatic hernia (CDH), 26 with meconium aspiration syndrome, and 5 with other diagnoses] were included in this study. Sixty-eight (75%) neonates survived. Fifty-two (76%) ECMO survivors developed CLD. There was no statistical difference between patients with and without CLD with regard to gender or gestational age. Patients with CLD had lower birth weight, were younger at the initiation of ECMO, and required longer ECMO runs. Patients with CDH developed CLD more often than infants with other underlying diseases (94 vs. 60%). Seventeen ECMO survivors (25%) developed severe CLD.

**Conclusion:**

The incidence of CLD after neonatal ECMO is substantial. Risk factors for its development include CDH as an underlying condition, the necessity for early initiation of ECMO, and the need for ECMO over 7 days.

## Introduction

Neonatal extracorporeal membrane oxygenation (ECMO) is an extracorporeal technique, which provides support to critically ill neonates suffering severe respiratory and/or cardiac failure refractory to conventional treatment with a high likelihood of mortality and a potentially reversible etiology.

Severe respiratory failure still remains the main indication for neonatal ECMO. The most common underlying diseases include congenital diaphragmatic hernia (CDH), meconium aspiration syndrome (MAS), and persistent pulmonary hypertension (PPHN) (1).

The application of ECMO has improved the survival of neonates with severe respiratory failure but carries also a higher risk of long-term morbidity. Among neonatal ECMO survivors, long-term pulmonary sequelae are well described (2–6). The development of chronic lung disease (CLD) may be an early marker for future pulmonary morbidity (7). Obstructive patterns with bronchospasm, asthma, and decreased exercise tolerance are the most common conditions of long-term respiratory morbidity (8).

The aim of this study was to assess the incidence and severity of CLD in neonates after ECMO in patients treated in our institution, distinguished by the primary underlying condition. Furthermore, we aimed to identify perinatal characteristics associated with the development of CLD.

## Materials and Methods

### Subjects

Neonates who received support with ECMO between January 2019 and October 2021 were selected from the neonatal intensive care unit (NICU) of the Department of Neonatology of the University Children’s Hospital Mannheim, University of Heidelberg. The indication criteria for and the allocation to ECMO were based on the recommendations of the Extracorporeal Life Support Organisation (ELSO) (9) for all the underlying diagnoses, with the exception of CDH. ECMO initiation in neonates with CDH was based on the recommendations made by CDH EURO Consortium Consensus Guideline Update 2015 (10). All patients received veno-arterial ECMO. All gestational ages were included. Exclusion criteria were congenital heart defects (except patent ductus arteriosus and persistence of foramen ovale) and inborn errors of metabolism. This study was approved by the local ethics committee of the Medical Faculty Mannheim of the University of Heidelberg.

### Chronic Lung Disease

The diagnosis of CLD was made as reported before (10, 11): if there was an additional need for oxygen supplementation on day 28 after birth, CLD was diagnosed. Target values for oxygen saturation at the moment of diagnosis were ≥92%. An oxygen reduction test was performed on day 28 of life to check the need for oxygen administration in not mechanically ventilated patients. The severity of CLD was differentiated into three grades according to the additional need for oxygenation at day 56 after birth or at discharge, whichever point came first: mild CLD with no need for supplemental inspired oxygen (fraction of inspired oxygen [FiO_2_] ≤ 0.21), moderate CLD (FiO_2_, 0.22–0.29), and severe CLD (FiO_2_ ≥ 0.30 and/or positive pressure).

### Data Collection

Demographic, pre- and perinatal, as well as clinical and laboratory data, were collected from the patients’ records, including demographic variables, diagnosis, prenatal parameters (if available), referral from another institution, highest oxygenation index prior to ECMO, age at initiation of ECMO, duration of ECMO, total duration of mechanical ventilation, duration of oxygen dependency, and type of respiratory support at discharge.

### Statistical Methods

Statistical analysis was performed with SAS Version 9.4 (SAS Institute Inc., United States). Descriptive statistics were used to describe the demographic characteristics of the patients and the incidence of CLD. Neonates with CLD and neonates without CLD were compared with regard to gender, birth weight, prematurity, oxygenation index (OI) prior to ECMO, age at onset of ECMO, duration of ECMO, and underlying diagnosis. The chi-square or Fisher’s exact test for categorical variables and the *t*-test or the Mann–Whitney U test for quantitative variables were applied to determine statistical differences between the two groups. For normally distributed variables, results were compared through means and standard deviations; when variables were not normally distributed, comparisons were made through medians and ranges. The development of CLD was also analyzed depending on the underlying condition. Logistic regression analyses were used to identify predictors of the development of CLD. A *p*-value of 0.05 or less was considered significant.

## Results

### Demographic and Clinical Characteristics of the Study Cohort

Between January 2019 and October 2021, 93 neonates received ECMO support within the first week of life at the Neonatal ECMO Center of University Children’s Hospital Mannheim. Complete data for analysis were available for 91 patients. Fifty-three neonates were born at our institution, whereas 38 patients (42%) were referred specifically to receive ECMO support. Severe respiratory failure was the indication for neonatal ECMO in all patients, CDH being the most common underlying disease (66%), followed by MAS (29%). Sixty-eight patients survived to be discharged (75%). CLD was present in 76% of ECMO survivors. Seventeen of these patients (33%) developed severe CLD, and 21 and 46% developed moderate and mild CLD, respectively. Patients with CDH developed CLD more often than infants with other underlying diseases (94 vs. 60%). Severe CLD was present in 42% of the CDH patients whereas only 7% of patients with other underlying conditions developed severe CLD. Eleven surviving patients (16%) needed some kind of respiratory support at discharge, and three patients (4%) were discharged with invasive ventilation. The perinatal characteristics of the study population and its respiratory outcome, distinguished by the primary underlying condition are shown in Table 1.

**TABLE 1:** Clinical perinatal characteristics of the patients treated with neonatal extracorporeal membrane oxygenation (ECMO).

	All patients	CDH	MAS	Other
Subjects, n (male)	91 (49)	60 (30)	27 (15)	5 (4)
Preterm (<37 weeks)	7	7	0	0
Inborn	53	53	0	0
Age at ECMO-Onset, h	24 (3–146)	19 (3–146)	32 (10–113)	45 (27–66)
Duration of ECMO, days	9 (1–28)	9 (1–28)	9 (4–14)	10 (7–14)
Survivors, n	68	38	26	4
CLD	52	34	16	2
Mild CLD	24	8	16	0
Moderate CLD	11	11	0	0
Severe CLD	17	15	0	2
RS at discharge	11	9	0	2
Oxygen	1	0	0	1
HFNC	6	5	0	1
NIV	1	1	0	0
IV	3	3	0	0

*Data are expressed as median (range) or n. Total group of patients and subgroups according to diagnosis are shown. CLD, chronic lung disease; CDH, congenital diaphragmatic hernia; ECMO, extracorporeal membrane oxygenation; HFNC, high-flow nasal cannula; IV, invasive ventilation; MAS, meconium aspiration syndrome; NIV, non-invasive ventilation; OI, oxygenation index; CLD, chronic lung disease; RS, respiratory support.*

### Comparison of All Patients With and Without Chronic Lung Disease

Patients who developed CLD did not differ from those who did not in neither in gender nor gestational age. Patients with CLD had lower birth weight and OI prior to ECMO, they were younger at the initiation of ECMO and required longer ECMO runs. The same results were found when comparing patients with severe CLD and no CLD (Table 2). Place of birth was statistically significantly different between patients with and without CLD. Since our institution is a referral center for patients with CDH and especially severe CDH are already diagnosed before birth, this condition cannot be considered a risk factor for the development of CLD.

**TABLE 2:** Comparison of clinical perinatal characteristics between patients with and without chronic lung disease.

	No CLD	CLD^α^	*P*-value	Severe CLD^α^	*P*-value
Subjects (male)	14 (7)	52 (28)	0.7100	17 (10)	0.6200
Preterm (<37 weeks)	0	3	1.0000	1	1.0000
Birth weight, g*	3581 (±480)	3107 (±424)	0.0006	3022 (±537)	0.0050
Inborn	1	31	0.0008	13	0.0001
Age at ECMO-Onset, h^+^	28.5 (10–66)	19 (3–146)	0.0360	14 (3–48)	0.0320
Duration of ECMO, days^+^	7.0 (4–13)	8.5 (5–16)	0.0490	10 (6–16)	0.0380
Highest OI prior to ECMO*	45 (±10)	36 (±15)	0.0440	34 (±14)	0.0330
Diagnoses					
CDH	2	34	0.0010	15	<0.0001
MAS	10	16		0	
Other	2	2		2	

Data are expressed as mean (standard deviation)*, median (range)^+^, or n. Shown are comparisons from perinatal parameters between subgroups according to the presence and severity of chronic lung disease. ^α^Patients with CLD and with severe CLD were compared with patients without CLD. CLD, chronic lung disease; CDH, congenital diaphragmatic hernia; ECMO, extracorporeal membrane oxygenation; MAS, meconium aspiration syndrome; OI, oxygenation index.

Consideration of all these perinatal characteristics showed good discrimination for predicting CLD in ECMO survivors [area under the curve (AUC) 0.86; *p* = 0.002]. Duration of ECMO was the only independent predictor of development of CLD (odds ratio = 1.39; 95% confidence interval = 1.02–1.90; *p* = 0.04). Seven days were established as the best cutoff point (sensitivity = 89%, specificity = 43%) for predicting CLD. An overview of all the perinatal characteristics of all patients with and without CLD is displayed in Table 2.

### Comparison of Congenital Diaphragmatic Hernia Patients With and Without Chronic Lung Disease

There was a disproportionate number of infants with CDH in the group of patients who developed CLD (65 vs. 14% *p* 0.001) and severe CLD (88 vs. 11% *p* < 0.0001) when compared with the group which did not develop CLD. Nevertheless, no statistically significant differences regarding perinatal characteristics could be found when comparing CDH patients with and without CLD. All three patients who needed invasive respiratory support at discharge had CDH as an underlying disease. A comparison of the perinatal characteristics between patients with CDH discharged with invasive ventilation and discharged without respiratory support showed no statistically significant differences.

### Comparison of Meconium Aspiration Syndrome Patients With and Without Chronic Lung Disease

Neonates with MAS developed no CLD (38%) or mild CLD (62%) after ECMO. When comparing neonates with MAS as an underlying condition, birth weight was the only perinatal characteristic that differed between the group with and without CLD: patients who developed CLD had lower birth weights (Table 3).

**TABLE 3:** Comparison of clinical perinatal characteristics in patients with meconium aspiration syndrome between those with and without chronic lung disease.

	No CLD	CLD	*P*-value
Subjects (male)	10 (4)	16 (11)	0.23
Preterm (<37 weeks)	0	0	
Birth weight, g*	3666 (±537)	3273 (±307)	0.03
Inborn	0	0	
Age at ECMO-Onset, h^+^	23 (10–37)	27.5 (10–113)	0.28
Duration of ECMO, days^+^	6 (4–13)	9 (5–14)	0.14
Highest OI prior to ECMO*	44 (±8)	45 (±15)	0.87

Data are expressed as mean (standard deviation)*, median (range)^+^, or n. Shown are subgroups according to the presence of chronic lung disease. CLD, chronic lung disease; ECMO, extracorporeal membrane oxygenation; MAS, meconium aspiration syndrome; OI, oxygenation index.

## Discussion

Neonatal ECMO has improved the survival of neonates with severe respiratory failure (2, 5, 11). However, improved survival might carry a higher risk of long-term morbidity among survivors depending on several factors, such as underlying conditions (7, 12). MAS, PPHN, and neonatal respiratory distress syndrome were historically the most common indications for neonatal ECMO (8). Due to perinatal care improvement, their incidence declined and to date, CDH represents one-third of neonatal ECMO diagnoses (8). Irrespective of the underlying disease, the development of CLD represents an important risk factor for impaired pulmonary outcomes in neonatal ECMO survivors (13).

Mechanical ventilation and supplemental oxygen contribute to the pathogenesis of CLD (2). The use of protective ventilation, and avoiding barotrauma and hyperoxia during the course of ECMO may mitigate ventilation-induced lung injury and may promote lung healing until the underlying disease heals. On the other hand, only critically ill neonates who otherwise would have died receive support with ECMO, so a poor respiratory outcome would be expected in these patients. In a prospective study of 219 neonates who met the criteria for ECMO, Vaucher et al. (14) showed that ECMO survivors had a 50% reduction in the incidence of CLD compared with patients with severe respiratory failure who survived after conventional or high-frequency ventilation. In a randomized controlled trial of 78 1-year-old infants after severe respiratory failure, Beardsmore et al. (15) showed that ECMO does not prevent sequelae of severe respiratory disease in the newborn period, but that respiratory function following ECMO was no worse and indeed appeared slightly better than following conventional treatment. Our institution is a national referral center for neonatal ECMO and neonates with CDH. In our study, the incidence of CLD after neonatal ECMO was clinically relevant (76%) and much higher than those reported in previous studies (14, 16). The number of infants with CDH in the group of patients who developed CLD was disproportioned (65 vs. 14%). The underlying condition may be an important factor associated with pulmonary morbidity after neonatal ECMO (7, 12) and the high prevalence of patients with CDH in our study population (56% diagnosis among ECMO survivors) may explain our high incidence of CLD. We found a significantly higher rate of CLD in patients with CDH than in all other patient populations, which is in accordance with previous studies (2, 14, 17) and comprehensible, since infants with other diagnoses (such as MAS or pneumonia) have a normal lung development, while the pathophysiology of CDH includes lung hypoplasia and PPHN. Furthermore, CDH patients that require ECMO concern the most severe cases (18). ECMO can rescue these severely ill patients, providing time for improvement of pulmonary hypertension and avoiding iatrogenic lung injury (10) until the surgery can be performed, but the sequelae of the severe pulmonary hypoplasia remain present, compromise the lung function in these patients, and contribute to a prolonged need of oxygen and invasive ventilation. Using data from the CDH Study Group registry, Van Den Hout et al. (19) described an incidence of CLD of 41% in patients with CDH. Similar results were found in a study of 255 patients with CDH conducted in our center: CLD was found in 45% of the subjects. However, the incidence of CLD in patients with CDH not treated with ECMO was 28% vs. 94% in those that required ECMO (20).

Within our population of neonates with severe respiratory failure that required ECMO support, patients who developed CLD and severe CLD had lower birth weights, were younger at the initiation of ECMO, and required longer ECMO runs compared to patients who did not develop CLD. Since hemodynamic problems and not only respiratory failure may be relevant for the ECMO initiation in neonates with CDH, the higher rate of CLD in neonates with CDH and a large number of CDH patients in our study population may explain the earlier ECMO requirement with lower oxygenation indexes in patients who developed CLD.

Of all perinatal characteristics examined, the duration of ECMO was the only parameter identified as an independent predictor of the development of CLD.

If patients with CDH and lung hypoplasia were excluded, the incidence of CLD was 60%. All these patients were outborn neonates with severe respiratory failure, who were referred to our institution when they met the inclusion criteria for ECMO. In contrast with Schwendeman et al. (21), we did not find differences between neonates with and without CLD regarding age at initiation of ECMO or highest oxygenation index prior to ECMO. Birth weight was the only perinatal characteristic that differed between the two groups. In our study, no MAS patients developed moderate or severe CLD. Different studies have evaluated long-term pulmonary sequelae in patients with an underlying condition different from CDH. A cross-sectional study conducted by Boykin et al. (22) in 10–15-year-old patients who underwent ECMO after MAS, revealed air trapping and persistent airflow obstruction. Hamutcu et al. (4) also found a high prevalence of respiratory symptoms, lung hyperinsuflation, and airway obstruction in 48 ECMO survivors at age of 11 years. However, they found a direct correlation between the amount and duration of elevated oxygen exposure and barotrauma with the frequency of long-term respiratory complaints, suggesting that ECMO and lung-rest ventilation strategies do not prevent the development of pulmonary morbidity in later childhood, but may reduce its severity. These findings could be in accordance with the lower incidence of CLD and no incidence of severe CLD found in our patients with a diagnosis different from CDH and lung hypoplasia. Further studies correlating the development of CLD and its severity and long-term pulmonary morbidity through childhood and age in patients with a diagnosis different from CDH following neonatal ECMO should be performed in order to verify this hypothesis.

In our study, nearly all CDH patients (94%) developed CLD after ECMO (22% mild, 30% moderate, and 42% severe). Nine patients with CDH (24%) needed respiratory support at discharge: five infants were discharged with a high-flow nasal cannula (HFNC), one with non-invasive ventilation (NIV) and three (8% CDH survivors) required long-term tracheostomy and invasive home mechanical ventilation. Our incidence of tracheostomy in CDH survivors is in line with those described in prior publications (17). In patients with CDH, the need for ECMO has been associated with the degree of long-term pulmonary morbidity with an increased likelihood of tracheostomy placement (22, 23). While prenatal risk stratification identifies severe cases, its correlation with long-term respiratory morbidity remains unknown (24) and postnatal identification of infants with irrecoverable lung hypoplasia that will develop severe CLD remains difficult (21). A few CDH patients without CLD made it impossible to identify statistically significant differences concerning perinatal characteristics when comparing CDH patients with and without CLD. However, the differences found when comparing patients with all diagnoses (early ECMO requirement despite lower oxygenation indexes and longer ECMO runs) might identify the most severe cases of CDH patients with a higher risk to develop CLD.

Respiratory long-term outcome in terms of lung function and exercise capacity in CDH patients is well described in the literature (3, 4, 12, 13, 15, 22, 25, 26). Although CLD is a risk factor for the development of pulmonary morbidity in later childhood, only few studies have described the incidence and severity of CLD following neonatal ECMO (14, 16), infants with CDH being unrepresented in those studies. Due to several factors such as lung hypoplasia and the need for prolonged ventilation and high oxygen supply in their neonatal period, infants with CDH are at risk of developing CLD (27). The development of CLD with a need for respiratory support on day 30 of life has shown to be a strong independent predictor of long-term morbidity (not only pulmonary but also developmental and in terms of future readmissions and surgical procedures) in patients with CDH (28), we believe that is very important to be aware of the very high incidence of CLD after ECMO in these patients in order to optimize their long-term care and the counseling of their families.

### Limitations

Our study has several limitations: it is a retrospective, observational, single-center study. The high incidence of CLD and severe CLD in neonates with CDH made it impossible to identify statistically significant differences concerning perinatal characteristics when comparing CDH patients with and without CLD and we only found differences, when comparing patients with all diagnoses. However, since the underlying condition may be an important factor associated with pulmonary morbidity after neonatal ECMO, patients with different diagnoses should be analyzed separately. Because of the small number of patients, it was also impossible to identify perinatal factors associated with the need for respiratory support at discharge. A multicenter study with a greater number of patients should be performed in order to identify related perinatal characteristics for the development of CLD and the need for home mechanical ventilation, distinguished by the primary underlying condition.

## Conclusion

The incidence of CLD after neonatal ECMO in this study was clinically relevant. CDH as an underlying condition, the necessity for early initiation of ECMO, and the need for ECMO over 7 days are factors associated with its development. Nearly all our patients with CDH requiring ECMO support developed CLD, one-third developed severe CLD, and 24% needed respiratory support at discharge. Since the development of CLD has shown to be a strong predictor of long-term morbidity in patients with CDH, these findings should be considered in order to optimize the long-term care of these patients.

## Data Availability Statement

The original contributions presented in this study are included in the article/supplementary material, further inquiries can be directed to the corresponding author.

## Ethics Statement

The studies involving human participants were reviewed and approved by the Local Ethics Committee of the Medical Faculty Mannheim of the University of Heidelberg. Written informed consent from the participants’ legal guardian/next of kin was not required to participate in this study in accordance with the national legislation and the institutional requirements.

## Author Contributions

AP, AG, FD, SH, and NR contributed to the concept and design, acquisition, interpretation of data, and drafting of the article. TD and TS contributed to the interpretation of data and revised the article for important intellectual content. All authors approved the final version of the article.

## Conflict of Interest

The authors declare that the research was conducted in the absence of any commercial or financial relationships that could be construed as a potential conflict of interest.

## Publisher’s Note

All claims expressed in this article are solely those of the authors and do not necessarily represent those of their affiliated organizations, or those of the publisher, the editors and the reviewers. Any product that may be evaluated in this article, or claim that may be made by its manufacturer, is not guaranteed or endorsed by the publisher.
